# Comparison of sediment biomarker signatures generated using time-integrated and discrete suspended sediment samples

**DOI:** 10.1007/s11356-024-32533-5

**Published:** 2024-02-26

**Authors:** Hari Ram Upadhayay, Steven J. Granger, Adrian L. Collins

**Affiliations:** Net Zero and Resilient Farming, Rothamsted Research, North Wyke, Okehampton, EX20 2SB UK

**Keywords:** Source fingerprinting, Alkanes, Fatty acids, Sediment, Biotracers

## Abstract

Sediment source fingerprinting using biomarker properties has led to new insights in our understanding of land use contributions to time-integrated suspended sediment samples at catchment scale. A time-integrated mass-flux sampler (TIMS; also known as the ‘Phillips’ sampler), a cost-effective approach for suspended sediment collection in situ. Such samplers are widely being used to collect sediment samples for source fingerprinting purposes, including studies using biomarkers as opposed to more conventional tracer properties. Here, we assessed the performance of TIMS for collecting representative sediment samples for biomarkers during high discharge events in a small lowland grassland-dominated catchment. Concentrations of long odd-chain n-alkanes (> C_23_) and both saturated free and bound fatty acids (C_14_-C_32_), as well as compound-specific ^13^C were compared between sediment collected by both TIMS and autosamplers (ISCO). The results showed that concentrations of alkanes, free fatty acids, and bound fatty acids are consistently comparable between TIMS and ISCO suspended sediment samples. Similarly, compound-specific ^13^C signals were not found to be significantly different in the suspended sediment samples collected using the different samplers. However, different magnitudes of resemblance in biomarker concentrations and compositions between the samples collected using the two sediment collection methods were confirmed by overlapping index and symmetric coordinates-based correlation analysis. Here, the difference is attributed to the contrasting temporal basis of TIMS (time-integrated) vs. ISCO (discrete) samples, as well as potential differences in the particle sizes collected by these different sediment sampling methods. Nevertheless, our findings suggest that TIMS can be used to generate representative biomarker data for suspended sediment samples collected during high discharge events.

## Introduction

Excessive suspended sediment in aquatic ecosystems can have significant impacts on their water quality and integrity (Bilotta and Brazier 2008, Yi et al. 2008). Human activity, particularly land use change, combined with the increasing occurrence of extreme precipitation, has caused a significant increase in soil erosion and sediment delivery to many aquatic systems (Owens 2020). Elevated suspended sediment concentrations (SSC) contribute directly to the degradation of aquatic systems through reductions in ecosystem productivity resulting from elevated turbidity and concomitant decreased light transmission through the water column (Walling and Collins 2016) and, indirectly, via associated nutrients and contaminants which bind to fine-grained sediments causing additional reductions in water quality.

Although much is known about the soil erosion processes and rates that occur in agricultural and forest systems (Labrière et al. 2015; Montgomery 2007), attention has shifted to understanding the relative contributions of difference land use types to total suspended sediment fluxes at the catchment scale (Collins et al. 2020; Collins et al. 2017). Here, improved understanding of which land uses are dominant in contributing to elevated sediment fluxes can support better targeting of management. Various tracers, such as radionuclides, stable isotopes, mineral magnetics, color, and biomarkers have been used to characterise sediments which, have in turn, led to new insights in the understanding of the contributions of the different sources areas of suspended sediment in catchments (Collins et al. 2020). These tracers can provide information on the delivery pathways and slope-to-channel connectivity at catchment scale (Upadhayay et al. 2020). Where tracers are applied using the sediment source fingerprinting approach, the contributing sediment source areas are deconvoluted using an unmixing model by comparing the composite tracers of the suspended sediment directly with those of the potential catchment sediment sources. The reliability and robustness of this approach, therefore, depend upon the collection of a representative suspended sediment sample, meaning that the sampling of suspended sediment for the analysis of tracers (i.e., fingerprint properties) is a critical task.

One widely used method for collecting suspended sediment samples is the deployment of a time-integrated mass-flux sampler (TIMS), also known as a ‘Phillips’ sampler (Phillips et al. 2000). The TIMS collects suspended sediment due to the large reduction in water flow velocity that occurs within it, compared to that of the watercourse. This is because the flow inlet of the sampler is far smaller than the sampler’s main chamber diameter. The sediment sample collected by the TIMS integrates a sample of the suspended sediment flux throughout the sampling period (low to high flows) and has been reported to collect representative suspended sediment samples in the case of geochemical, physical, and magnetic properties (Russell et al. 2000, Smith and Owens 2014), for diatom communities (Foets et al. 2020) and for quantifying suspended sediment transfer. One drawback to the TIMS, however, is that it has been shown to preferentially collect coarse sediment grains which can potentially lead to an underestimation of the total suspended sediment flux at catchment scale (Perks et al. 2014, Smith and Owens 2014). Nevertheless, the TIMS is simple, cost-effective, and easy to deploy in a wide range of riverine environments and, as such, widely used to collect sediment for sediment source apportionment.

To date, no studies have examined whether sediment collected using TIMS is sufficiently representative for the application of biotracer in conjunction with the sediment source fingerprinting approach. This evidence gap is important since a growing number of source fingerprinting studies are applying biomarkers, as opposed to more conventional sediment properties (Gibbs 2008; Upadhayay et al. 2017; Collins et al. 2020). The potential underrepresentation of fine-grained sediment in samples collected using TIMS noted by previous studies could also create a bias when using biomarkers to trace suspended sediment sources. This is because biomarkers, like other tracers, tend to adsorb preferentially to the fine-grained particles (Upadhayay et al. 2020). Given this context, we present a detailed evaluation of the biomarker tracer composition of suspended sediment collected using TIMS compared to that collected using a conventional autosampler in a field setting.

## Materials and methods

### Study catchment description

**Fig. 1:**
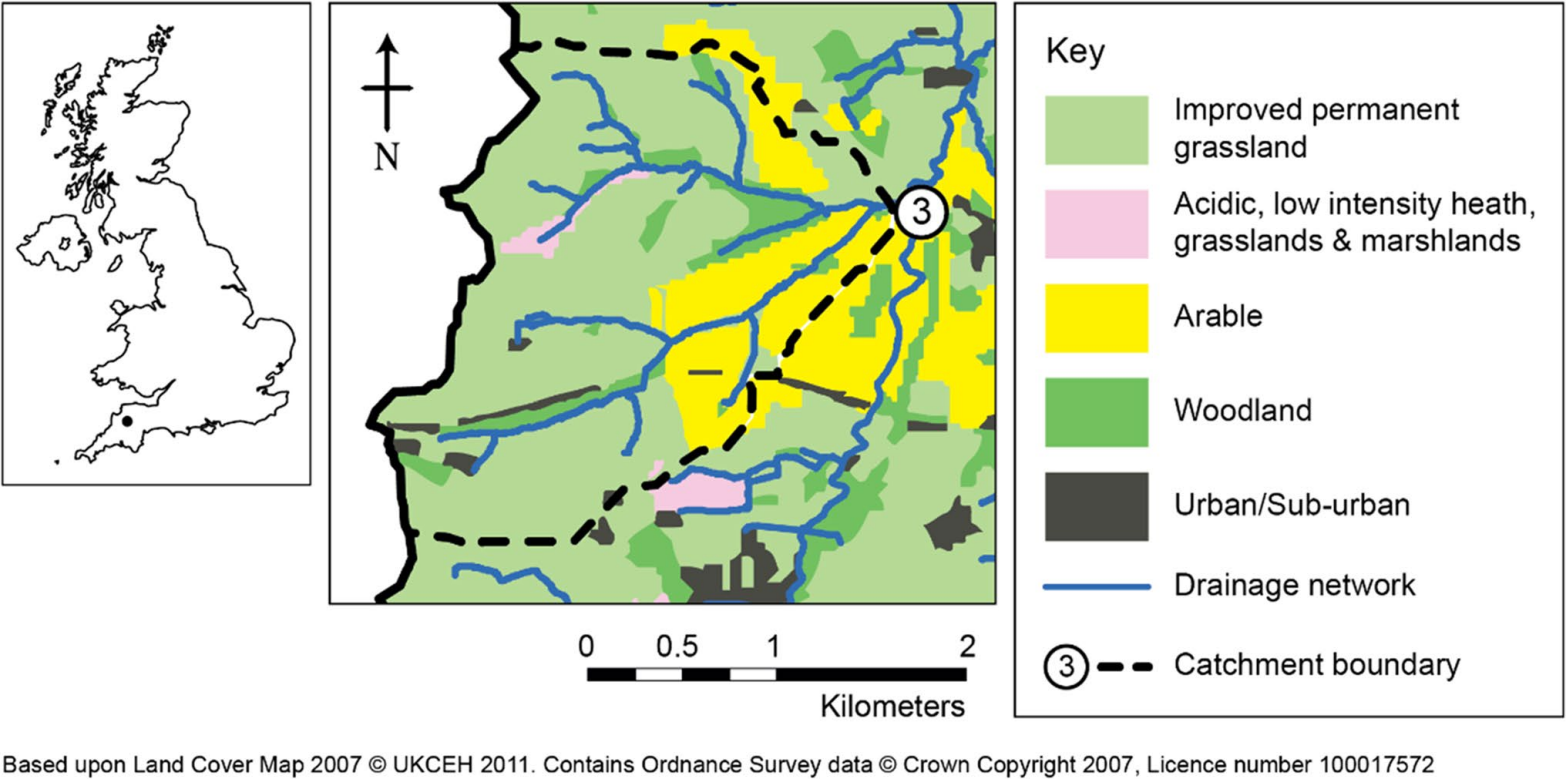
Location of the Upper River Taw Observatory within the UK and the land use in the nested sub-catchment (catchment 3)

The study was undertaken within the upper River Taw observatory (URTO), an instrumented catchment within the headwaters of the River Taw in southwest England (https://www.rothamsted.ac.uk/projects/upper-river-taw-observatory-urto) more details about which can be found in Granger et al. (2023). In short, the URTO consists of a 19-km stretch of the river that drains an area of 41.3 km^2^ which is monitored at the catchment outlet for discharge (Q) and various other physio-chemical parameters, including turbidity, on a 15-min timestep. Two further nested sub-catchments are monitored within the URTO which are 4.4 and 1.7 km^2^ in size. This study was undertaken using the 4.4 km^2^ catchment known locally as Lower Ratcombe and referred to as catchment 3 in Granger et al (2023) and hereafter (Fig. 1). River hydrology is primarily surface water driven and *Q* tends to be flashy in response to rainfall events while base flow is maintained during extended dry periods by water released through rock fissures. The soils of the study catchment are typically poorly draining, seasonally waterlogged clay-rich gley soils and brown earths and the dominant land use was improved grassland (71%) for animal grazing, but with a significant proportion of arable land (18%) and some woodland (10%) (Fig. 1).

### Storm event sampling

Storm events were targeted based on meteorological forecasts. Prior to a predicted event, two TIMS were placed in stream flow at the outlet to catchment 3. Sample lines of automated samplers (Teledyne ISCO, NE, U.S.A.) were also placed instream, and autosamplers were set to collect 1-L samples at timesteps of either 30 or 60 min depending on the expected duration and size of the forecast wet weather event. The autosamplers were also configured to ensure that sample collection occurred at the same time as a *Q* and turbidity measurement was taken. Once *Q* had dropped to a safe level, the TIMS were removed from the channel and their contents bulked into a collection barrel. Autosamplers were also stopped at this time and their samples taken back to the laboratory along with the TIMS samples.

### Sample processing

Once back at the laboratory, autosampler samples had a 250-mL sub-sample removed for measurement of various chemical and physical parameters including SSC through the filtration and subsequent drying at 105 °C of a known sample volume on a pre-weighed GF/C filter paper. The SSC data from these, and other sampled storm events at catchment 3, were then combined with the turbidity measurements recorded at those times to develop a calibration curve which enabled all recorded 15-min time step turbidity measurements to be converted to SSC. The remaining 750 mL of each autosampler sample was bulked in barrels. Both bulked samples were left for several days in a refrigerated environment to allow sediment to settle. Once the bulk of the sediment had settled to the bottom of the barrels, the remaining water was removed and passed repeatedly through a portable centrifuge to collect any remaining fine-grained particulate material. This material was then added to the previously separated sediment and water was further removed using a laboratory based static centrifuge until the material was about 500 mL in volume. This material was then frozen at − 20 °C and subsequently freeze dried before being sieved through a 32-µm mesh. The suspended sediment samples collected by the autosampler and TIMS are hereafter referred to as ISCO and TIMS sediment, respectively.

### Biomarker extraction and analysis

Bulk sediment carbon (C) and nitrogen (N) content and their stable isotope ratios were measured using a Carlo Erba NA2000 elemental analyzer (CE Instruments, Wigan, UK) interfaced with a PDZ Europa 20–22 isotope ratio mass spectrometer (SerCon Ltd., Crewe, UK). The isotopic results were expressed as natural abundance (*δ*) in parts per mil (‰) compared to international standards. The elemental and isotopic reference standard used was IAR001 (%N = 1.791; %C = 40.46; δ^15^N = 2.51‰; and δ^13^C =  − 25.99‰), a wheat flour standard sourced from Iso-Analytical, and calibrated against IAEA-N-1 and IAEA-CH6. The analytical precision for elemental and isotopic reference standards were 0.42% and 0.2‰ for C and 0.03% and 0.2‰ for N, respectively.

The detailed methodology for the biomarker extraction from sediment samples and subsequent analyses can be found in Upadhayay et al. (2022). Briefly, total free lipids (combined fatty acids (FA) and alkanes) were extracted from the sediment samples using dichloromethane to methanol ratio (9:1) by an accelerated solvent extraction machine (Donex 350) with three extraction cycles at 100 °C. Hydrolysable FAs (also known as bound fatty acids) were then released from solvent extracted residues (∼ 1 g; spiking with C_19_ FA) by treatment with 0.5 M KOH in methanol:water (9:1; 100 °C for 2 h) using a reflux method. The concentrations of alkanes were quantified using an Agilent 7890A GC with a flame ionization detector, whereas free (FFA) and bound (BFA) fatty acid concentrations were determined using an Agilent 6890 N/5973 N GC Mass Spectrometer. The reliability of the extraction process was checked by running a sediment sample spiked with an external standard containing FA C_19_ and alkane C_34_. The compound-specific δ^13^C signatures of alkanes, FFAs, and BFAs were determined using a Finnigan Mat 6890 GC coupled to a Finnigan Mat Delta Plus IRMS via a Combustion III interface and the δ^13^C was expressed relative to Vienna Pee Dee Belemnite (VPDB). The stability and linearity of the system were better than 0.06‰. The δ^13^C standard deviation from the standards was ± 0.35‰. The δ^13^C values of FAs were corrected for the contribution of δ^13^C values of the added methyl group during derivatisation. For the purposes of this study, we considered only long (> C_23_) odd-chain n-alkanes, saturated FFAs, and saturated BFAs (C_14_-C_32_) due to their relevance for sediment source apportionment using the fingerprinting approach (Collins et al. 2020; Upadhayay et al. 2017).

### Statistical analysis

A two-sample *t*-test was used to differentiate between ISCO and TIMS sediment for bulk C and N properties, biomarker content and compound specific δ^13^C. The overlapping index (similar area-under-the-curve of density distributions) was estimated (Pastore and Calcagnì 2019) for quantifying similarities or differences between biomarker/isotope distributions in TIMS and ISCO sediment samples. The overlapping index ranges from 0 to 1, where 1 represents ‘similar’ in terms of variable distribution and 0 indicates ‘distinct.’ This index does not assume the normality of distributions nor any other distributional form and works properly even in the presence of multimodality (Pastore and Calcagnì 2019). Besides absolute biomarker concentrations, biomarker data were also considered in terms of their composite nature as each biomarker is part of the whole and provides relative information. Therefore, symmetric coordinates (a specific type of log-ratio transformation) (Kynčlová et al. 2017; Reimann et al. 2017) were calculated before correlation analysis, which was conducted separately for alkanes, FFAs, and BFAs of the ISCO and TIMS sediment. This approach was adopted since it addresses the potential for the hidden influence of unaccounted biomarkers in the composition. All statistical analysis were performed in R (R Core Team 2022) using packages “robComposition” (Templ et al. 2011) and “Overlapping” (Pastore 2018). All figures for presenting results were designed using the package “ggplot2” (Wickham 2009).

## Results and discussion

### Event characteristics

**Fig. 2:**
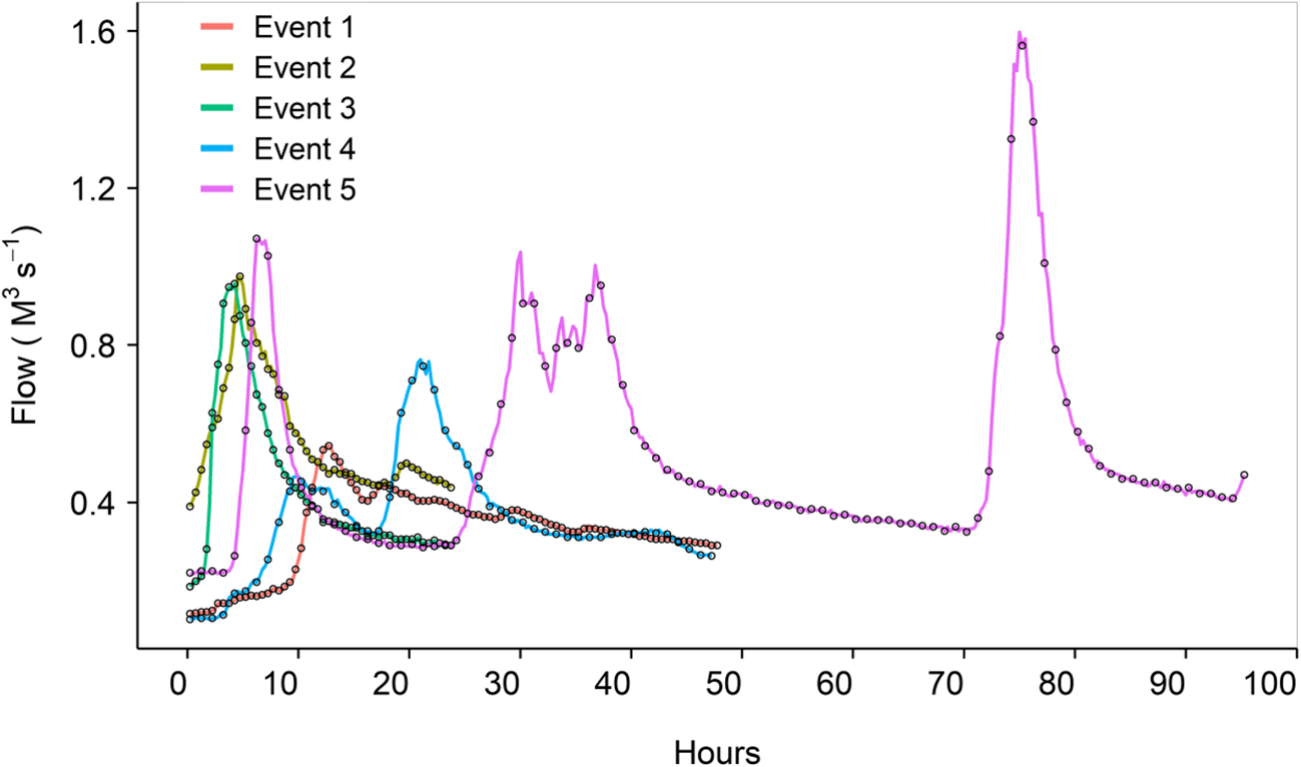
Hydrograph of five high storm events used to collect sediment by deploying ISCO and TIMS in Lower Ratcombe stream. Open circles indicate ISCO sampling times

Summary data for the five events reported in this study are contained within Table 1. The five storm events differed in magnitude with peak recorded *Q* ranging between 0.5 and 1.6 m^3^ s^−1^; the smallest event being Event 1 and the largest Event 5. While higher *Q* is typically associated with higher SSC, this was not observed in the case of the study events. While Event 5 had the highest recorded peak *Q* and the highest recorded peak SSC, events with lower peak *Q* values sometimes had higher SSC concentrations than those events with higher peak *Q* (e.g., Events 3 and 4). These hydro-sedimentological responses can be due to a number of different factors such as land cover and use, antecedent soil moisture, and rainfall intensity, all of which affect soil erosion and sediment connectivity to the stream channel (Upadhayay et al. 2022). Events 1 to 3 represent simple hydrographs (Fig. 2) with rapidly rising *Q* in response to rainfall and a most attenuated decrease in *Q*. Events 4 and 5, however, are multi-peaked hydrographs (Fig. 2) representing periods of time where *Q* rises and falls in response to different periods of rainfall. In all cases, peak SSC occurred on, or just before peak *Q*.

**Table 1:** Summary data for the five storm events sampled using both ISCO and TIMS sampling methodologies

Event ID	Date	Sampling duration	Flow (m^3^ s^−1^)	SSC (mg L^−1^)	Event suspended sediment load (t)	ISCO sampler frequency	Sediment sample mass (g)		Event load sampled by TIMS (%)
							ISCO	TIMS	
1	06/11/2018	47 h 30 min	0.1–0.5	0–128	1.3	30 min	1.6	2.1	1.6 × 10^–4^
2	18/12/2018	23 h 30 min	0.4–1.0	9–101	1.5	30 min	1.3	2.4	1.6 × 10^–4^
3	12/03/2019	23 h 30 min	0.2–1.0	6–490	3.9	30 min	-	-	-
4	25/10/2019	47 h 0 min	0.1–0.8	4–546	6.3	1 h	3.2	11.4	1.8 × 10^–4^
5	13/01/2020	95 h 0 min	0.2–1.6	6–826	18.9	1 h	4.6	12.7	6.7 × 10^–5^

The masses of material collected by the TIMS and ISCO sampling approaches were measured simply by measuring the mass of material left after freeze drying. Typically, the mass of material collected by each approach increased with the increasing load of suspended sediment transported during each event.

### Bulk sediment data

**Fig. 3:**
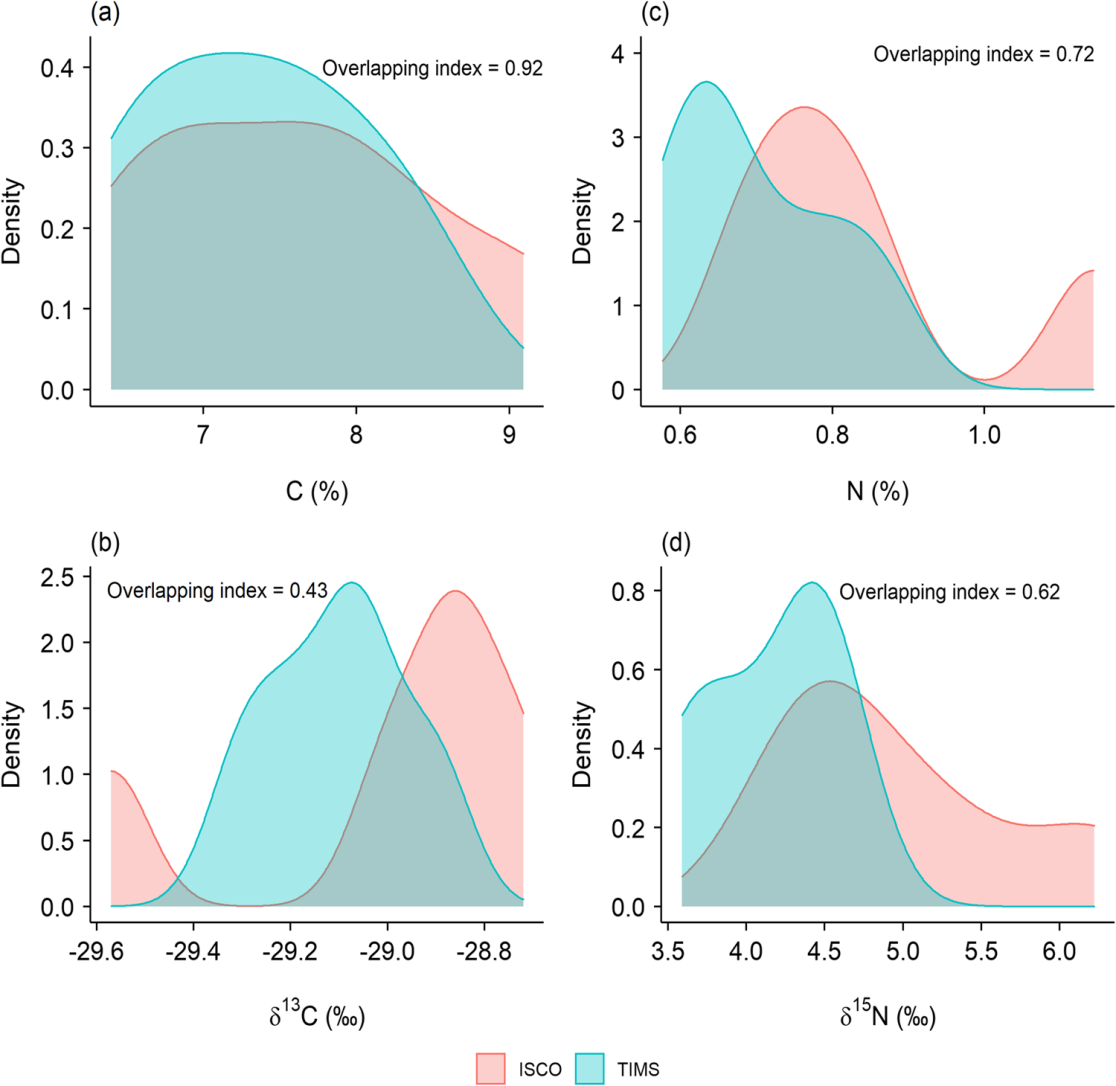
Comparisons of the density distributions of carbon (**a**) and nitrogen (**c**) content and their respective isotope values (**b**, **d**) in the ISCO and TIMS sediment samples, using the overlapping index

The C and N content of the suspended sediment samples collected using the ISCO (7.6 ± 1.0% and 0.8 ± 0.2%, respectively) and TIMS (7.3 ± 0.8% and 0.7 ± 0.1, respectively) were not statistically different. However, when the data was compared using the overlap of the area-under-the-curve of density distributions (Fig. 3), differences were more apparent. While the C content of suspended sediment collected using the two different methods appears similar (overlapping index = 0.92), the N content of the samples was less so (overlapping index = 0.72) (Fig. 3a, c). While there is no evidence in the literature pertaining to differences in the bulk N content of suspended sediment collected using these different methods, researchers have reported that sediment collected using TIMS compared to other sampling approaches can have both similar (Russell et al. 2000) and dissimilar (Keßler et al. 2020) bulk C contents. The two different sediment samples were also not significantly different in the case of their δ^13^C and δ^15^N signatures. However, values of 0.43 and 0.62 for the overlap index for the density distributions of δ^13^C and δ^15^N values (Fig. 3b, d), respectively, suggest that the isotopic values in sediment collected using the two different approaches differed, possibly due to the corresponding differences in the temporal basis of the samples (i.e., time-integrated vs. discrete) as well as potential differences in the particle size distributions of the different samples.

### Compound-specific signatures

#### Comparison of general concentrations

The results for the concentrations of alkanes, FFA, and BFA in the suspended sediment samples collected using the ISCO and TIMS generally showed no significant differences (Table 2). Concentrations of BFA C_26_ and C_28_ were, however, significantly higher in sediment collected using the TIMS compared to the ISCO. Despite the similarity of the alkane, FFA, and BFA concentrations between the TIMS and ISCO sediment samples, compounds were found to differ when examined using the overlap of the area-under-the-curve of density distribution. More specifically, for alkanes, the overlap ranged between 0.49 and 0.82; for FFAs, the overlap was slightly higher, ranging between 0.65 and 0.92, while the overlap range for BFA was extremely wide ranging from 0.19 to 0.82.

**Table 2:** Distribution of biomarkers content and their δ^13^C values in the ISCO and TIMS sediment samples

Compound	C-chain length	Content (µgC/g sediment)		Overlapping index	δ^13^C (‰)		Overlapping index
		ISCO	TIMS		ISCO	TIMS	
Alkanes	C_23_	1.0 ± 0.2	1.2 ± 0.4	0.82	− 32.4 ± 1.3	− 32.4 ± 2.0	0.39
	C_25_	3.4 ± 1.6	3.2 ± 0.6	0.71	− 32.1 ± 1.5	− 32.2 ± 1.3	0.83
	C_27_	8.5 ± 3.1	8.4 ± 1.1	0.71	− 32.3 ± 1.3	− 32.5 ± 1.4	0.85
	C_29_	11.0 ± 3.3	10.6 ± 1.3	0.49	− 34.8 ± 1.6	− 34.9 ± 1.5	0.96
	C_31_	9.1 ± 2.7	8.4 ± 1.3	0.78	− 35.9 ± 1.1	− 36.2 ± 1.7	0.82
	C_33_	3.8 ± 1.3	3.2 ± 0.5	0.73	− 35.3 ± 1.5	− 36.3 ± 0.6	0.55
Free fatty acids	C_14_	10.0 ± 4.9	5.5 ± 2.9	0.65	− 29.3 ± 1.3	− 30.6 ± 0.6	0.49
	C_16_	51.6 ± 16.1	36.6 ± 11.1	0.59	− 30.1 ± 1.0	− 30.4 ± 0.4	0.84
	C_18_	51.4 ± 30.2	34.5 ± 20.9	0.65	− 31.5 ± 1.1	− 31.5 ± 0.4	0.65
	C_20_	12.7 ± 4.1	10.3 ± 3.4	0.78	− 33.5 ± 0.7	− 34.2 ± 0.3	0.53
	C_22_	23.0 ± 3.8	21.2 ± 3.7	0.85	− 34.4 ± 0.8	− 34.8 ± 0.4	0.78
	C_24_	28.5 ± 3.9	26.8 ± 3.7	0.89	− 34.3 ± 0.6	− 34.9 ± 0.3	0.49
	C_26_	38.1 ± 6.7	35.5 ± 5.2	0.92	− 35.5 ± 0.6	− 35.6 ± 0.3	0.81
	C_28_	35.5 ± 4.5	36.0 ± 5.1	0.91	− 35.5 ± 0.5	− 35.5 ± 0.3	0.87
	C_30_	25.5 ± 3.9	26.0 ± 3.4	0.92	− 36.4 ± 0.4	− 36.4 ± 0.4	0.88
	C_32_	11.5 ± 2.5	11.5 ± 1.8	0.70	− 37.6 ± 0.5	− 37.5 ± 0.4	0.65
Bound fatty acids	C_14_	16.3 ± 4.9	12.7 ± 4.2	0.82	− **32.3 ± 0.6**	− **33.7 ± 0.8**	0.42
	C_16_	94.9 ± 12.9	99.3 ± 45.7	0.63	− 31.5 ± 0.8	− 32.5 ± 0.6	0.39
	C_18_	40.0 ± 5.2	45.9 ± 21.1	0.62	− 32.1 ± 0.4	− 31.9 ± 0.4	0.83
	C_20_	8.3 ± 1.1	9.4 ± 0.8	0.41	− 34.1 ± 0.8	− 33.8 ± 0.7	0.78
	C_22_	14.4 ± 2.7	19.6 ± 5.4	0.37	− 34.0 ± 0.5	− 34.1 ± 0.4	0.56
	C_24_	10.8 ± 2.0	14.1 ± 2.5	0.19	− 34.7 ± 0.2	− 35.0 ± 0.9	0.58
	C_26_	**8.2 ± 2.0**	**11.1 ± 1.7**	0.56	− 35.2 ± 0.5	− 34.9 ± 0.5	0.88
	C_28_	**7.2 ± 1.7**	**10.1 ± 1.4**	0.47	− 34.6 ± 0.5	− 34.9 ± 0.4	0.87
	C_30_	3.1 ± 0.8	4.4 ± 0.9	0.68	− 35.0 ± 0.7	− 35.5 ± 0.9	0.72
	C_32_	1.2 ± 0.2	1.6 ± 0.5	0.28	− 32.0 ± 0.2	− 32.7 ± 0.7	0.16

Bold figures indicate significantly different at *α* = 0.05

#### Compound-specific n-alkanes

**Fig. 4:**
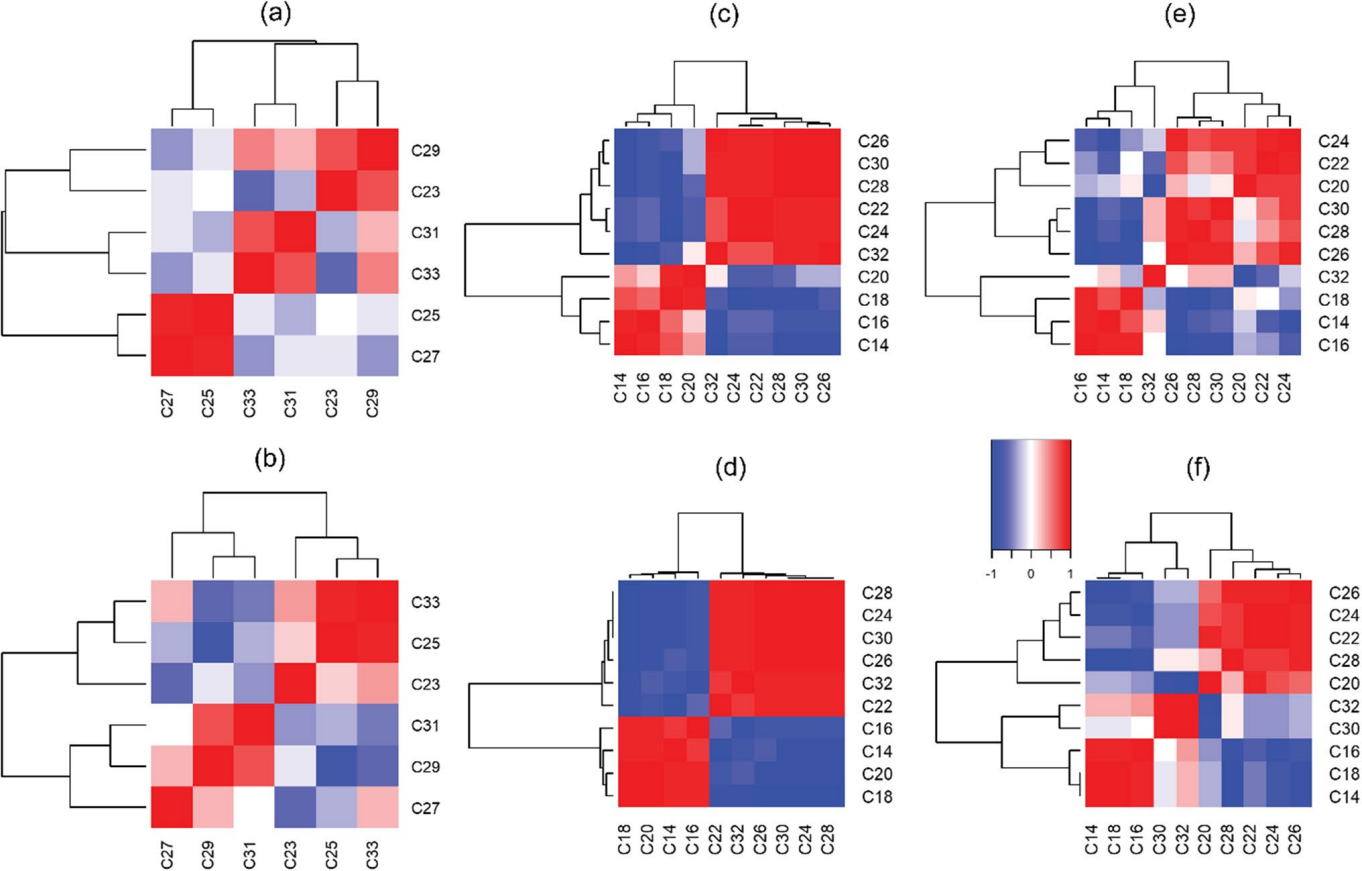
Heat-map of correlations based on symmetric coordinates for the alkane (**a**, **b**), free fatty acid (**c**, **d**), and bound fatty acid (**e**, **f**) data for the ISCO (upper panel) and TIMS (lower panel) sediment samples. Biomarkers along the axes are sorted according to the results of the cluster analysis

Alkanes are neutral lipids derived from plant waxes with different numbers of C atoms in the molecules that are indicative of different origins. Long-chain (> C_27_) n-alkanes are derived from the waxes of terrestrial plants (Chikaraishi and Naraoka 2003), medium-chain length (C_21_-C_25_) n-alkanes are produced by lower plants and aquatic macrophytes (Tolosa et al. 2013), while short chain-length (C_15_-C_19_) n-alkanes are typically derived from aquatic algae (Bianchi and Canuel 2011). Differences in the n-alkane composition between the ISCO and TIMS sediment samples are illustrated in Fig. 4a, b. We observed strong correlations of n-alkanes, with three clusters in the ISCO samples (C_27_/C_25_, C_33_/C_31_, and C_23_/C_33_) but only two major clusters (C_27_/C_29_/C_31_ and C_23_/C_25_/C_33_) in the TIMS sediment samples. This indicates that the ISCO and TIMS sediment samples are different in terms of alkane composition. The study catchment is dominated by grassland and arable land uses (89%) (Fig. 1) and C_31_ and C_33_ n-alkanes have been reported to be dominant in such environments (Schäfer et al. 2016). This suggests that sediment collected by the ISCO sampling approach better represents the land use of the study catchment in terms of the composition of the n-alkane signature.

##### Compound-specific fatty acids

Differences also existed between the FFA composition of the ISCO and TIMS sediment samples (Fig. 4c, d). We observed two major clusters (≤ C_20_ and ≥ C_22_) in the ISCO and TIMS sediment samples consistent with their potential sources. Short-chain FFAs (≤ C_20_) are produced by all plants and also microorganisms, whereas long-chain FFAs (> C_22_) are mostly derived from vascular terrestrial plants (Chikaraishi 2014; Upadhayay et al. 2017), and therefore, long-chain FFAs are good indicators of terrestrial sediment sources. Long-chain FAs were more highly correlated with each other in the TIMS sediment samples compared to those collected using the ISCO, with two major clusters of FFA observed in both the TIMS and ISCO sediments (Fig. 4c, d). The strong correlation in long-chain FFA is consistent with the higher abundance of > C_22_ FFA reported in grass/arable land compared to deciduous forest (Schäfer et al. 2016; Zocatelli et al. 2012) and suggests that the TIMS collected a more representative sample of sediment than the ISCO in terms of FFAs. Such FFAs are relatively newly produced plant products and are delivered, along with fine-grained sediment, to watercourses.

Bound FAs, which represent FAs not extracted by the solvents used to extract FFAs, include both the breakdown products of other lipids and also previously FFAs which have become strongly associated with soil particles (Upadhayay et al. 2022). This BFA pool typically represents a relatively older FA pool than FFAs, often with a lower δ^13^C signature due to fractionations associated with FA cycling. Fatty acids can undergo selective microbial degradation in soil, such as odd-C numbered FAs produced by microbial α-oxidation of even-C numbered FAs (Matsumoto et al. 2007). Different clusters were observed in the BFAs compared to the FFAs for the ISCO and TIMS sediment samples (Fig. 4e, f). Importantly, the clusters found in the BFAs (C_16_/C_14_/C_18_, C_26_/C_28_/C_30_, and C_20_/C_22_/C_24_) were consistent with their potential land use sources in the study catchment. The results exhibited three clusters of BFAs based on correlation analysis in the TIMS sediment samples (C_14_/C_18_, C_20_/C_28_, and C_30_/C_32_) still consistent with their potential catchment sources. Overall, the C_26_ and C_28_ FA signatures in the TIMS sediment samples (Table 2) were found to be similar to those of the grassland surface soils (e.g., *C*_max_ is at C_26_ for long-chain fatty acids) close to the study catchment (unpublished data). This further suggests that TIMS can collect representative sediment-associated FA signatures in the study catchment.

The δ^13^C values of the n-alkanes, FFA, and BFA were not significantly different for the ISCO and TIMS sediment samples. The δ^13^C values of the biomarkers suggested that they originated from C3 plants (Matsumoto et al. 2007). However, although the δ^13^C values of the biomarkers in the TIMS and ISCO sediment samples were not significantly different, they were not highly similar based on the overlap of the area-under-the-curve of the corresponding density distributions. Here, the overlapping index ranges were 0.39–0.96, 0.49–0.88, and 0.16–0.88 for n-alkanes, FFAs and BFAs, respectively (Table 2). This clearly suggests that the δ^13^C distributions differ between the ISCO and TIMS sediment samples.

### Implications for sediment source assessment

 The mass of material collected by the ISCO autosamplers is dependent upon the sample frequency and volume. In our study, the time normalized mass of sediment collected by ISCO was between 28 and 38% less material than the TIMS (Table 1). This means that more material was available for the extraction of biomarkers and other analytes when sediment was sampled using the TIMS. This is one reason why TIMS have been adopted so widely for sediment source fingerprinting purposes (Collins and Walling 2004). One potential issue identified for the TIMS sampler, however, concerns the underrepresentation of the finest particles in the time-integrated sediment sample (Foets et al. 2020, Smith and Owens 2014), although, findings are contradictory in the sense that some researchers have reported similar particle size distributions in TIMS sediment samples compared with other samples (Goharrokhi et al. 2019).

Both biomarker content (Chen et al. 2016) and their stable isotope ratios (Upadhayay et al. 2022) have been used for sediment source apportionment. This study has shown that the biomarker content and the δ^13^C are not significantly different for sediment samples collected using the ISCO autosampler and the TIMS, but that the distribution of different biomarkers was often different (Table 2). Moreover, biomarker composition is not similar for the ISCO and TIMS sediment samples (Fig. 3) which may indicate biases in the different relative source contributions to the sediment collected by these different sample collection approaches. Therefore, researchers should be cautious when using different sediment sampling approaches when drawing conclusions on the sediment source area contributions using biomarkers and associated indices. To the best of our knowledge, this is the first study comparing biomarker contents and their stable isotope ratios in samples collected using ISCO and TIMS approaches. We inevitably must interpret our data based on the knowledge of what we would expect to find given the known potential sediment sources in the study catchment. Here, the n-alkane and FA concentrations and compositions in sampled sediment depends on the predominant vegetation type of the study catchment and the potential corresponding sediment sources therein. Based on the catchment land use information, we argue that TIMS can collect representative samples for generating sediment-associated biomarker signatures in the study catchment during the high discharge events responsible for soil erosion and sediment delivery, especially in the case of FFA and BFA. However, one potential issue with the TIMS (and indeed the ISCO) is that differences in the geochemical compositions of sediment collected in shallow and deep water using the sampler have recently been reported and attributed to hydrodynamic sorting (Lučić et al. 2021). Further research is therefore warranted to explore how the position of TIMS in the water column and channel cross-section, especially in larger river systems, impacts on the biomarker composition and compound-specific stable isotope values assembled for sediment samples.

## Conclusions

Alkanes and FAs are biomarkers with increasing adoption in sediment source apportionment studies for aquatic ecosystems. In this study, we have provided insights into the comparability of biomarker content and their ^13^C signals in sediment samples collected using an ISCO autosampler and a TIMS. We found that while biomarker content and the corresponding ^13^C signals were not significantly different in the sediment samples collected using the ISCO and TIMS approaches, biomarker distributions and compositional patterns were often not similar. Heterogeneity in biomarker composition might emerge in ISCO and TIMS sediment samples due to differences in the corresponding sediment sampling intervals. The sediment collected using an ISCO represents discrete sediment samples taken at a constant time interval in contrast to the TIMS which continuously samples sediment in situ throughout the period of deployment. More work is needed to explore the sensitivity of source apportionment estimates to the potential contrasts in biomarker signatures generated using different sediment sampling procedures. Overall, the TIMS was found to collect a representative sediment sample based on biomarkers content. As such, the use of TIMS to collect time-integrated sediment samples for analysis of biomarker signatures can broaden our knowledge of sediment sources in catchments impacted by various anthropogenic and natural perturbations.

## Data Availability

The datasets analysed in this study are available from the corresponding author on request.
